# Deuteros 2.0: peptide-level significance testing of data from hydrogen deuterium exchange mass spectrometry

**DOI:** 10.1093/bioinformatics/btaa677

**Published:** 2021-01-08

**Authors:** Andy M Lau, Jürgen Claesen, Kjetil Hansen, Argyris Politis

**Affiliations:** 1 Department of Chemistry, King’s College London, London SE1 1DB, UK; 2 Institute for Environment, Health and Safety, Microbiology Unit, SCK•CEN, Mol 2600, Belgium; 3 I-Biostat, Data Science Institute, Hasselt University, Hasselt 3500, Belgium

## Abstract

**Summary:**

Hydrogen deuterium exchange mass spectrometry (HDX-MS) is becoming increasing routine for monitoring changes in the structural dynamics of proteins. Differential HDX-MS allows comparison of protein states, such as in the absence or presence of a ligand. This can be used to attribute changes in conformation to binding events, allowing the mapping of entire conformational networks. As such, the number of necessary cross-state comparisons quickly increases as additional states are introduced to the system of study. There are currently very few software packages available that offer quick and informative comparison of HDX-MS datasets and even fewer which offer statistical analysis and advanced visualization. Following the feedback from our original software Deuteros, we present Deuteros 2.0 which has been redesigned from the ground up to fulfill a greater role in the HDX-MS analysis pipeline. Deuteros 2.0 features a repertoire of facilities for back exchange correction, data summarization, peptide-level statistical analysis and advanced data plotting features.

**Availability and implementation:**

Deuteros 2.0 can be downloaded for both Windows and MacOS from https://github.com/andymlau/Deuteros_2.0 under the Apache 2.0 license.

## 1 Introduction

Hydrogen deuterium exchange mass spectrometry (HDX-MS) is a structural MS technique which can be used to monitor changes in both protein structure and conformation (Marcsisin and Engen, 2010). HDX leads to increase in the protein mass which can be detected by high-resolution MS (Marcsisin and Engen, 2010). HDX-MS experiments are commonly performed in a differential manner which envisions that two or more datasets collected from the same protein but in different conditions are compared with one another to study changes in regional deuterium uptake as a function of environmental changes. Such changes can include the addition of interacting species such as substrates or even modifications to the target protein itself (Martens *et al.*, 2018).

Raw data acquired from HDX-MS must undergo several processing steps before it can be interpreted (Claesen and Burzykowski, 2017; Wales *et al.*, 2013). The first of these steps involves the identification of peptide ions from the raw MS spectra and calculation of their corresponding masses. The mass of a non-deuterated reference peptide is subtracted from its deuterated equivalent to determine the level of deuterium uptake. Finally, the deuterium uptake of each peptide is used to develop a representation of the protein conformation as a result of any changes in its structure and/or dynamics. For differential HDX-MS, data from a reference dataset is subtracted from a dataset belonging to an altered state, such as the protein in the absence or presence of a ligand. The significance of the changes identified from differential analyses can be evaluated using a range of statistical models including global threshold tests (Houde *et al.*, 2011), mixed effects models (Hourdel *et al.*, 2016) and hybrid significance tests (Hageman and Weis, 2019).While the first two steps of peptide identification and mass determination can be well managed using existing software such as ProteinLynx Global Server and DynamX (Waters Corporation), how the final step of statistical analysis and visualization is best performed is area of active discussion.

In 2019, we released our software Deuteros (Lau *et al.*, 2019) designed to perform rapid analysis and visualization of data from the Waters HDX-MS platform. Parallel to developments in the HDX-MS field, we present here Deuteros 2.0 which has been redesigned from the ground up with new features intended to further streamline the analysis of HDX-MS data.

## 2 How does it work?

### 2.1 Overview of Deuteros 2.0

Several notable improvements have been made during the redesign of Deuteros 2.0. First, the input data for Deuteros 2.0 takes the form of the DynamX ‘cluster’ file, replacing the ‘state’ and ‘difference’ files of the original Deuteros (Lau *et al.*, 2019). The cluster file contains a replicate-level breakdown of a HDX-MS dataset with the following hierarchy (highest level to lowest level): protein, state, peptide, timepoint, charge state and technical replicates. An advantage of using the cluster file is that an unlimited number of protein states can be included in a single input file, allowing greater data portability and facilitating better file management. Drop-down menus in Deuteros 2.0 allow users to easily select between proteins and states within the app, without having to switch between other input files, thus allowing for better time management. Users can optionally apply back-exchange correction to their data prior to downstream processing. To use this facility, users should acquire experimental data corresponding to maximally labeled peptides and supply this along with non-deuterated references as a control state to Deuteros 2.0. The control state is used to assess the degree of back exchange for each peptide and then to adjust the deuterium uptake of each peptide in the data intended for analysis, according to suggested correction procedures (Masson *et al.*, 2019).

**Fig. 1.**
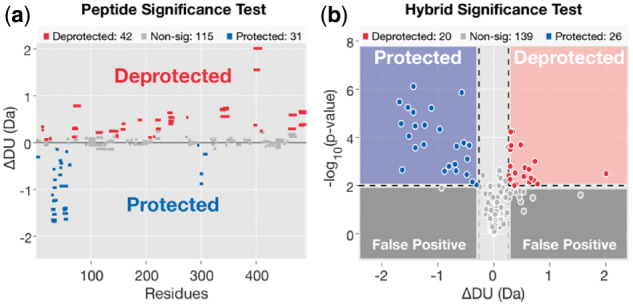
Statistical testing in Deuteros 2.0. Two statistical tests are offered in Deuteros 2.0: (**a**) peptide and (**b**) hybrid significance tests. Statistical models are applied to differential HDX-MS data to identify peptides which show statistically significant changes in deuteration between experimental states. Significant peptides are described as regions exhibiting deprotection or protection according to the direction of the deuteration difference. Data shown represent the XylE Δ(E397Q-WT) dataset from Martens *et al.* (2018)

The data imported into Deuteros 2.0 undergoes several transformations: (i) calculation of intensity-weighted average deuterium uptake and standard deviation—for each peptide, timepoint and state. An additional step removes data points belonging to redundant and/or poor-quality charge states; (ii) identification of common peptides between two user-selected states A and B (e.g. holo versus apo); (iii) calculation of deuterium uptake differences for each peptide and (iv) treatment of the uptake difference data with a user-selected statistical model to identify regions of the target protein exhibiting state-specific uptake patterns. For determining whether differences in deuterium uptake are statistically significant, Deuteros 2.0 offers a choice of two statistical models: a peptide-level significance test and a recently published hybrid significance test (Hageman and Weis, 2019) (Fig. 1).

To aid data interpretation, we have expanded the interface of Deuteros 2.0 with statistics-enabled facilities for evaluating the kinetics plot of individual peptides. For differential HDX-MS, we have included several graphical data presentation formats including Woods, volcano and barcode plots. Deuteros 2.0 is also compliant with community guidelines on HDX-MS data reporting by automatically producing a summary of data quality for the imported dataset (Masson *et al.*, 2019).

### 2.2 Peptide-level significance testing

The peptide-level significance test uses multiple regression (Kutner, 2005) to fit deuterium uptake kinetics to a linear model (Equation 1).

(1) $$D_{ij}=\beta_{0}+\beta_{s}s_{i}+\beta_{t}t_{j}+\beta_{st}\left(s_{i}t_{j}\right)+\varepsilon_{ij}$$

where $D_{ij}$ is the deuterium content of a peptide from state$\;s_{i}$, at labelling time $t_{j}$ and residual $\varepsilon_{ij}\;∼\;N(0,\sigma_{ij}^{2})$. The variables $s_{i}$ and $t_{j}$ are categorical and indicate, respectively, to which state peptide *i* belongs, and at which time point $D_{ij}\;$is measured. The linear model tests if changes in the deuterium content of the peptide are associated with changes in the protein state ($\beta_{s}$), the deuteration time point ($\beta_{t}$), or both ($\beta_{st}$). Additionally, the probability of detecting false positives is controlled by the Benjamini–Hochberg procedure for multiple testing (Benjamini and Hochberg, 1995). This False Discovery Rate controlling method adjusts the *P*-value of each test using m, the number of hypotheses tested and i the ordered rank of the *P*-value (Equation 2):

(2) $$p^{*}=p\times (m/i)$$

The null hypothesis is rejected on the basis that the adjusted *P*-value is less than the significance level. To provide users with flexibility with comparing different statistical models, we have included a recently devised hybrid significance test (Hageman and Weis, 2019). Briefly, the hybrid significance test is a two-pronged statistical test which first evaluates whether the deuterium uptake difference of a peptide between two states is greater than a threshold value calculated to a user-defined significance level. A Welch’s *t*-test is then used to confirm the significance (Hageman and Weis, 2019).

### 2.3 Data visualization

Deuteros 2.0 improves on the original Deuteros software by including several redesigned and new data visualization methods. These include improvements to the ‘Coverage Plot’ section of the software through the addition of comparative facilities allowing users to quickly determine changes in coverage and redundancy between experiments. We anticipate that this feature will extend the usefulness of Deuteros 2.0 in aiding users with making experimental design choices, such as evaluating whether or not a change in digestion enzyme improves coverage to certain regions of targets. We foresee that this feature may be particularly useful for studies on difficult targets such as membrane proteins.

Under the ‘Advanced Plot’ section, users can assess data through one of the statistics-enabled plots. These include the Woods and volcano plots. Woods plots depict each peptide as a horizontal bar with length equivalent to the peptide length and are particularly useful for data presentation as they additionally depict both coverage and redundancy. The volcano plot format visualizes deuterium uptake differences along the horizontal axis and statistical significance on the vertical axis. Peptides with significant positive changes in deuterium uptake (areas deprotected upon change), significant negative changes (areas protected upon change) and non-significant changes follow a red, blue and gray color scheme, respectively.

Data processed and statistically treated using Deuteros 2.0 can be further visualized on molecular structures. Using this feature, protein data bank (PDB) files in either PyMOL (Schrödinger, 2015) or Chimera (Pettersen *et al.*, 2004) can be formatted to highlight regions of the protein identified through Deuteros 2.0 to be statistically significant. Coverage, redundancy and significant peptide regions determined through Woods or volcano plots are enabled for exporting to molecular graphics.
